# gExcite: a start-to-end framework for single-cell gene expression, hashing, and antibody analysis

**DOI:** 10.1093/bioinformatics/btad329

**Published:** 2023-05-23

**Authors:** Linda Grob, Anne Bertolini, Matteo Carrara, Ulrike Lischetti, Aizhan Tastanova, Christian Beisel, Mitchell P Levesque, Daniel J Stekhoven, Franziska Singer

**Affiliations:** ETH Zurich, NEXUS Personalized Health Technologies, 8952 Schlieren, Switzerland; SIB Swiss Institute of Bioinformatics, Zurich, Switzerland; ETH Zurich, NEXUS Personalized Health Technologies, 8952 Schlieren, Switzerland; SIB Swiss Institute of Bioinformatics, Zurich, Switzerland; ETH Zurich, NEXUS Personalized Health Technologies, 8952 Schlieren, Switzerland; SIB Swiss Institute of Bioinformatics, Zurich, Switzerland; Department of Biomedicine, University Hospital Basel and University of Basel, 4031 Basel, Switzerland; Department of Dermatology, University of Zurich, University of Zurich Hospital, 8952 Schlieren, Switzerland; Department of Biosystems Science and Engineering, ETH Zurich, 4058 Basel, Switzerland; Department of Dermatology, University of Zurich, University of Zurich Hospital, 8952 Schlieren, Switzerland; ETH Zurich, NEXUS Personalized Health Technologies, 8952 Schlieren, Switzerland; SIB Swiss Institute of Bioinformatics, Zurich, Switzerland; ETH Zurich, NEXUS Personalized Health Technologies, 8952 Schlieren, Switzerland; SIB Swiss Institute of Bioinformatics, Zurich, Switzerland

## Abstract

**Summary:**

Recently, CITE-seq emerged as a multimodal single-cell technology capturing gene expression and surface protein information from the same single cells, which allows unprecedented insights into disease mechanisms and heterogeneity, as well as immune cell profiling. Multiple single-cell profiling methods exist, but they are typically focused on either gene expression or antibody analysis, not their combination. Moreover, existing software suites are not easily scalable to a multitude of samples. To this end, we designed gExcite, a start-to-end workflow that provides both gene and antibody expression analysis, as well as hashing deconvolution. Embedded in the Snakemake workflow manager, gExcite facilitates reproducible and scalable analyses. We showcase the output of gExcite on a study of different dissociation protocols on PBMC samples.

**Availability and implementation:**

gExcite is open source available on github at https://github.com/ETH-NEXUS/gExcite_pipeline. The software is distributed under the GNU General Public License 3 (GPL3).

## 1 Introduction

Recently, Cellular Indexing of Transcriptomes and Epitopes by Sequencing (CITE-seq) was introduced as a droplet-based single-cell profiling technology that enables the analysis of Antibody Derived Tags (ADTs) in addition to the gene expression (GEX) readout (Stoeckius *et al.* 2017). Taken together, CITE-seq can provide information on both the mRNA and surface protein level for the same cell. Unlike bulk approaches, it offers insights on the single-cell level, allowing unprecedented insights in e.g. cell differentiation, immune compartment, and tumor heterogeneity (Zhu *et al.* 2017, Kulkarni *et al.* 2019). Moreover, hashing antibodies that tag ubiquitously expressed surface markers can be utilized for cell barcoding, allowing the multiplexing of samples (Stoeckius *et al.* 2018). A variety of methods exists for the analysis of scRNA-seq data, including the widely used Seurat (Hao *et al.* 2021) and Scanpy (Wolf *et al.* 2018) suites, as well as pipelines such as scAmpi (Bertolini *et al.* 2022) and CReSCENT (Mohanraj *et al.* 2020). In addition, methods for either CITE-seq analysis or hashing demultiplexing are available, e.g. Seurat (Hao *et al.* 2021), totalVI (Gayoso *et al.* 2021), and CiteFuse (Kim *et al.* 2020).

However, these tools typically require pregenerated expression counts and do not offer integrated solutions for these early and typically resource-intensive steps such as the read mapping. In addition, existing tools require at least a basic knowledge of a scripting language like R or Python in order to perform a single-cell data analysis.

Thus, despite the availability of tools for individual analysis steps, a start-to-end pipeline would simplify multimodal single-cell analysis. To this end, we implemented gExcite (pipeline for Gene Expression and CITE-seq analysis), a workflow that facilitates hashing demultiplexing, and individual as well as combined ADT and GEX analysis from raw reads up until readily interpretable output such as cluster and cell type identification with combined information on gene and protein expression. Moreover, gExcite offers a customizable template for GEX and ADT-based differential expression analysis. All steps of the workflow are embedded into the Snakemake workflow manager (Mölder *et al.* 2021), following the latest Snakemake best practices. Thereby, gExcite provides the functionality to scale the analysis to many samples in parallel and make seamless use of high-performance computing infrastructure. Moreover, gExcite does not require particular knowledge of any scripting language to be run. Taken together, gExcite presents a scalable and reproducible pipeline for multimodal single-cell analysis.

## 2 Workflow

**Figure 1.**
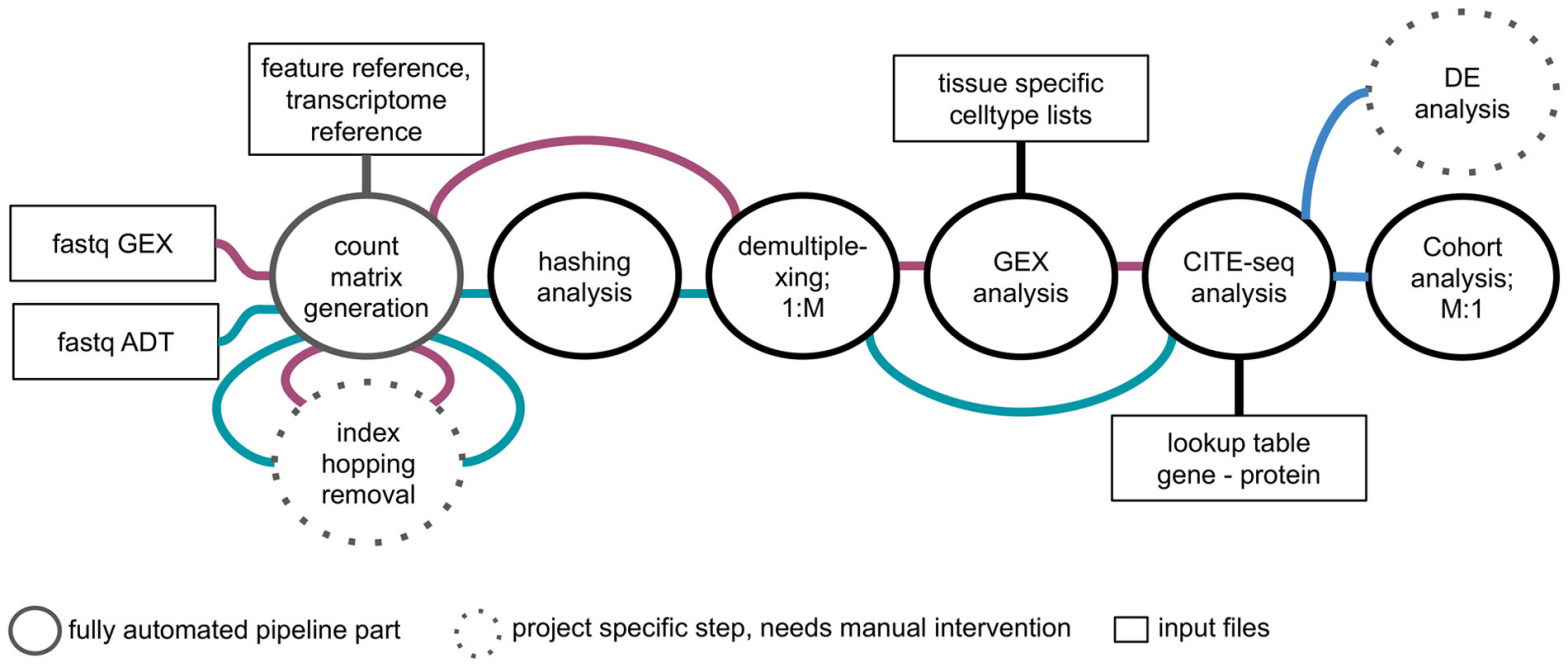
Overview of the workflow implemented in gExcite starting from raw fastq GEX and ADT files up until a combined analysis of GEX and ADT.

The workflow implemented in gExcite can be conceptually divided as shown in Fig. 1.

### 2.1 Read mapping

Raw reads are mapped to a reference genome using 10× Genomics Cell Ranger software (Zheng *et al.* 2017). GEX and ADT libraries are processed independently to infer read counts per gene per cell, or read counts per antibody per cell, respectively.

If required by the experimental setup (e.g. sequencing on the NovaSeq without dual indexing), gExcite provides functionality to perform index-hopping removal using the DropletUtils package (Griffiths *et al.* 2018), resulting in a clean count matrix that can be further processed.

### 2.2 Hashing

Cell hashing allows for multiplexing, facilitates doublet detection and allows superloading of cells in single-cell transcriptomics experiments (Stoeckius *et al.* 2018).

For demultiplexing, first CITE-seq-Count (Mimitou *et al.* 2019) is applied to count hashtags per cell. Subsequently, normalization, demultiplexing, and removal of doublets as well as negatives are performed using the Seurat framework (Hao *et al.* 2021). In addition, gExcite offers normalization options where the counts are normalized across cells and hashtags independently, and the resulting hashtag assignments are compared (refer to Supplementary Material S1 for details).

### 2.3 GEX analysis

gExcite includes the scRNA-seq analysis pipeline scAmpi as a Snakemake module to analyze GEX data, including among other analyses cell type assignment, clustering, and pathway enrichment analysis. Importantly, the quality control functionality of scAmpi is used to further filter the GEX data and to remove contamination, such as likely empty droplets or ambient RNA (Bertolini *et al.* 2022).

### 2.4 CITE-Seq analysis

In the previous GEX analysis step, the GEX data have been filtered as part of the quality control. Only cells present in both GEX and ADT data are preserved for downstream analyses. Thus, the ADT data are filtered based on the results of the GEX quality control.

gExcite offers functionality to analyze and visualize antibody expression data on its own as well as in combination with the corresponding gene expression (Supplementary Fig. S2A, panel C and D). The link between antibodies and corresponding genes is provided by the user with a gene to protein dictionary. Gene and antibody expression counts can be simultaneously visualized in a Uniform Manifold Approximation and Projection (UMAP) plot (Supplementary Fig. S2A, panel A). The UMAP embedding is computed either based on gene expression, antibody expression, or the combination thereof (Supplementary Fig. S2B). This allows the assessment of cell similarity based on the different data types. BremSC (Wang *et al.* 2020) is utilized to provide a combined gene and antibody-based clustering (Supplementary Fig. S2A, panel B). Note that for CITE-seq analysis raw counts can contain background noise from ambient antibodies and nonspecific antibody binding. gExcite allows setting an experiment-specific threshold per antibody to accommodate for this noise (Supplementary Fig. S2A, panel D and F). Cells exceeding the threshold are labeled as positive for the respective antibody. To aid manual threshold definition and to ease interpretation of antibody expression, gExcite provides cell type-expression ridge plot visualizations (Supplementary Fig. S2A, panel E and F).

Additionally, gExcite provides functionality for differential expression analysis on GEX and ADT data, as well as to collectively analyze samples multiplexed within one experiment. More information can be found in Supplementary Material S3.

## 3 Conclusion and outlook

The proposed workflow offers comprehensive functionality for the automated analysis of both GEX and ADT, as well as hashed single-cell data. It facilitates an easy yet in-depth quality control of the analyzed samples as well as supports the interpretation of single-cell experiments.

Key aspects are its flexibility, ease-of-use, and scalability, which allows the reproducible application also to large-scale data sets.

## Supplementary Material

btad329_Supplementary_Data

## Data Availability

The example data underlying this article are part of the data set available on EGA under accession EGAS00001005849 on https://ega-archive.org/studies/EGAS00001005849. Conflict of interest: The authors have no conflict of interest to declare.
